# Mouse corticospinal system comprises different functional neuronal ensembles depending on their hodology

**DOI:** 10.1186/s12868-019-0533-5

**Published:** 2019-09-23

**Authors:** Rafael Olivares-Moreno, Mónica López-Hidalgo, Alain Altamirano-Espinoza, Adriana González-Gallardo, Anaid Antaramian, Verónica Lopez-Virgen, Gerardo Rojas-Piloni

**Affiliations:** 1 Departamento de Neurobiología del Desarrollo y Neurofisiología, Instituto de Neurobiología, Universidad Nacional Autónoma de México, Campus UNAM-Juriquilla, Querétaro, Mexico; 2 Escuela Nacional de Estudios Superiores, Juriquilla, UNAM, Universidad Nacional Autónoma de México, Campus UNAM-Juriquilla, Querétaro, Mexico

**Keywords:** Sensorimotor cortex, Layer 5, Pyramidal tract neurons, GCaMP, Calcium imaging, Neural circuits

## Abstract

**Background:**

Movement performance depends on the synaptic interactions generated by coherent parallel sensorimotor cortical outputs to different downstream targets. The major outputs of the neocortex to subcortical structures are driven by pyramidal tract neurons (PTNs) located in layer 5B. One of the main targets of PTNs is the spinal cord through the corticospinal (CS) system, which is formed by a complex collection of distinct CS circuits. However, little is known about intracortical synaptic interactions that originate CS commands and how different populations of CS neurons are functionally organized. To further understand the functional organization of the CS system, we analyzed the activity of unambiguously identified CS neurons projecting to different zones of the same spinal cord segment using two-photon calcium imaging and retrograde neuronal tracers.

**Results:**

Sensorimotor cortex slices obtained from transgenic mice expressing GCaMP6 funder the Thy1 promoter were used to analyze the spontaneous calcium transients in layer 5 pyramidal neurons. Distinct subgroups of CS neurons projecting to dorsal horn and ventral areas of the same segment show more synchronous activity between them than with other subgroups.

**Conclusions:**

The results indicate that CS neurons projecting to different spinal cord zones segregated into functional ensembles depending on their hodology, suggesting that a modular organization of CS outputs controls sensorimotor behaviors in a coordinated manner.

## Background

The sensorimotor cortex (primary somatosensory and motor cortices) plays a fundamental role in movement execution by means of the long-range projection of its pyramidal tract neurons (PTNs) to several subcortical structures. PTNs are thick-tufted cells located in layer 5B. PTNs differ from intratelencephalic pyramidal neurons, which are slender-tufted cells located in layer 5A and project mainly to the striatum and contralateral cortex [1–3].

Thick-tufted PTNs comprise a heterogeneous class of cells that project subcortically to the spinal cord, posteromedial thalamic nucleus, superior colliculus, pontine nucleus, red nucleus and striatum [4–8]. Sensorimotor cortex layer 5 (L5) neurons projecting to different targets have electrophysiological and morphological [5, 7] characteristics that are target-related, suggesting that they form segregated classes of neurons [6, 8–10]. Moreover, PTNs show a partial segregation within L5B. For example, the location of corticotectal cells is more superficial than that of corticopontine and corticotrigeminal neurons [5, 9]. Moreover, recently two types of PTNs have been identified with distinct gene expression: one type projects mainly to the thalamus and the other to motor centers in the medulla. Both types of PTNs have specialized roles in motor control and are segregated into L5B [10].

In particular, PTNs projecting to the spinal cord comprise a complex multifunctional system involved in sensorimotor integration [11, 12]. In this way, the CS descending projection modulates spinal cord pre-motor interneurons [13–17] but also modulates sensory information [18–20], thus playing a major role in voluntary motor control [21, 22]. In the rat, CS neurons activate at different time latencies, distinct dorsal horn and intermediate zone neurons of the same segment of the spinal cord [23]. Moreover, skilled movements are controlled differentially by distinct CS circuits [24]. This suggests that PTNs are organized into hierarchical subsystems that control sensorimotor integration through different outputs.

To understand whether CS neurons are functionally organized depending on their hodology, we analyzed the co-activation of spontaneous calcium transients, in identified corticospinal PTNs of the mouse sensorimotor cortex. Our results show that the functional synchronization between CS neurons projecting to the dorsal horn and to ventral zones, of the same segmental target, is higher within the cells projecting to the same area, suggesting that the CS system is organized into functional ensembles depending on their hodology.

## Materials and methods

Adult (P65-P70) transgenic mice (C57BL/6J-Tg (Thy1-GCaMP6f) GP5.1Dkim/J; The Jackson Laboratory) expressing GCaMP6f under the Thy1 promoter were used. All animals were housed in a temperature-controlled (24 °C) colony room and maintained in a 12-h/12-h light/dark cycle (lights on at 7:00 a.m.). Food and water were provided ad libitum.

### Retrograde labeling of corticospinal projection neurons

Mice were initially anesthetized in a hermetic box (4% isoflurane mixed with O_2_) and placed in a stereotaxic frame (WPI 502063). Then, isoflurane was lowered (1–1.5%) to maintain the anesthesia during the surgical procedure. Body temperature was maintained using a thermostatically regulated heating pad. All surgical procedures were performed under sterile conditions. A laminectomy was performed followed by an incision of the dura to expose cervical (C4–C5) spinal cord segments. FluoroGold (FG) (Fluorochrome; 4% in distilled water) and cholera toxin subunit b (ChT) recombinant conjugated with Alexa 594 (Molecular Probes; 1 mg/mL in PBS) were injected into the dorsal horn (DH) and intermediate gray matter and ventral horn (IVH) of the same segment (FG in the DH and ChT-594 for the IVH) of the spinal cord. The DH injections were made 700 µm lateral to the midline, and the tip of the pipette was set at a depth of 100 µm. The IVH injections were made 600 µm lateral to the midline, and the tip of the pipette was set at 1000 µm depth. Discrete injections (30–50 nL in 10 min) were performed by means of pressure pulses (200 ms; 30 PSI). The pipette tip was kept for 3 min after the end of each injection. The wound was closed with absorbable sutures. The animals received a single dose of metamizole (300 mg/kg SC) and then allowed to recover from. During pos-operative period, the animals were continually monitored to observe any symptom of pain or discomfort.

### Histology

The distribution of CS neurons was analyzed in 3 experiments. To do so, 5 to 8 days after the tracer injections, the animals were deeply anesthetized (pentobarbital 45 mg/kg i.p.) and perfused through the heart with phosphate-buffered saline (PBS) 0.1 M, followed by paraformaldehyde (4% in 0.1 M of PBS, pH 7.4). The brains and spinal cord were removed and stored overnight in vials containing the same fixative. Coronal brain sections were cut using a vibratome (Leica VT1200S) at 50-μm intervals. Slices were immunolabeled to amplify the FG signal and count FG-labeled cells. Hence, slices were permeabilized and blocked for 2 h at room temperature in 0.5% Triton x-100 (Sigma Aldrich X100-100ML) diluted in 100 mM PBS containing 4% normal goat serum (Gibco). Primary antibody was diluted (1:1500 Rabbit anti-FluoroGold, Fluorochrome) in PBS containing 1% NGS for 48 h at 4 °C. Secondary antibody (1:500 goat anti-Rabbit Alexa-488 molecular probes #A21235) was incubated for 2–3 h at room temperature in PBS containing 3% NGS and 0.3% Triton X-100.

### Confocal imaging

For detection of CS cells, mosaic images covering ~ 3.0 × 3.0 mm were acquired with a confocal microscope (Zeiss 780 LSM) using a Zeiss LD PCI Plan-Apochromat 25×/0.8 ImmKorr DIC multimersion objective with a spatial resolution of 0.554 µm per pixel. For simultaneous imaging of the fluorophores, the following settings were used: Alexa-488 (Argon-laser 488 nm, detection range: 502–533 nm), Alexa-594 (DPSS laser 561 nm, detection range: 502–594 nm), FG (DPSS laser 561 nm, detection range: 568–594 nm). The neuron density was computed using Amira software 5.6.0. To do that, we first manually select the soma location of the retrogradely labeled CS neurons from the confocal images. Then, using Amira’s function *profile along axis*, the neurons were counted in 50 μm bins along the cortical layers.

### Slice preparation

Five to eight days after FG and ChT injections, mice were deeply anesthetized with isoflurane (5% isoflurane mixed with O_2_) in an induction chamber for 5 min. Animals were euthanized by decapitation after making sure the animal was completely unresponsive to tail pinch. The brain was quickly removed and placed in ice-cold artificial cerebrospinal fluid cutting solution (ACSF) constantly bubbled with carbogen (95% O_2_ and 5% CO_2_). The ACSF contained (in mM): NaCl 92, KCl 2.5, NaH_2_PO_4_ 1.2, NaHCO_3_ 30, HEPES 20, d-glucose 25, sodium ascorbate 5, thiourea 2, sodium pyruvate 3, MgSO_4_ 10 and CaCl_2_ 0.5 (pH 7.4). The brain was glued rostral-end upward onto an agar block and mounted on a vibratome (Leica VT1200S). Coronal slices (300 μm-thick) containing sensorimotor cortex (− 1.2–2.1 mm from Bregma) were obtained. The slices were kept in ACSF (in mM): *N*-methyl-d-glucamine 93, KCl 2.5, NaH_2_PO_4_ 1.2, NaHCO_3_ 30, HEPES 20, d-glucose 25, sodium ascorbate 5, thiourea 2, sodium pyruvate 3, HCl 93, MgSO_4_ 10 and CaCl_2_ 0.5 bubbled in carbogen at 32–34 °C for at least 30 min. After the recovery time, the slices were moved to a holding solution containing (in mM): NaCl 92, KCl 2.5, NaH_2_PO_4_ 1.2, NaHCO_3_ 30, HEPES 20, d-glucose 25, sodium ascorbate 5, thiourea 2, sodium pyruvate 3, MgSO_4_ 2 and CaCl_2_ 2. For calcium imaging, the slices were kept in a recording solution containing (in mM): NaCl 124, KCl 2.5, NaH_2_PO_4_ 1.2, NaHCO_3_ 24, HEPES 5, d-glucose 12.5, MgSO_4_ 2 and CaCl_2_ 2 [25]. The temperature was maintained at 30 ± 1 °C with a temperature controller (VWR Standard heat block #13259-030).

### Two-photon calcium imaging

Two-photon calcium imaging of the deeper L5 pyramidal cells (~ 800–1000 µm under the pia) was performed with an upright microscope (Zeiss 780 LSM) coupled to a Chameleon femtosecond-pulse laser (Coherent, Chameleon Ultra) to excite GCaMP6f (920 nm) and FG dye (800 nm). Fluorescence was detected using a non-descanned detector (NDD, Carl Zeiss) with a Zeiss 25× multi-immersion objective (LD PCI Plan-Apochromat 25×/0.8 ImmKorr DIC M27). Time series calcium images were collected at 5 Hz with ZEN software (ZEN 2012 v8.1) at a spatial resolution of 1.661 µm per pixel. The laser power was adjusted for each slice but never above 50 mW. The gain of the detector was usually adjusted to improve the signal-to-noise ratio and avoid pixel saturation.

### Analysis of sensorimotor cortex functional imaging

Ongoing calcium fluctuations of corticospinal (FG- and ChT-positive cells) L5 neurons were acquired for 4 min. Image processing was carried out with Image J (v.1.42q, National Institutes of Health) and custom routines written in MATLAB. Regions of interest (ROIs) were determined using a semi-automated algorithm provided by Pnevmatikakis et al. [26]. Briefly, the cell bodies of all active neurons in a field of view were identified by denoising the spatiotemporal fluorescence up to a desired voxel-dependent noise level and inferring compact neuron shapes with fluorescence dynamics [26]. After that, the time series of each ROI was computed as DF/F, where F is the fluorescence intensity at any frame and DF in the difference between F and the resting fluorescence of each neuron. Recordings were visually inspected and the cells with an unstable baseline (continuous decay or increased fluorescence) were excluded from the analysis (21 out of 240 cells).

To determine the level of synchronization of neuronal calcium activity, the co-activation of calcium signals was computed between all pairs of hodologically identified neurons (CS projecting to dorsal horn: FG-positive or CS projecting to the intermediate gray matter: ChT-positive). Calcium events were determined from the derivative of the calcium activity. Only the events in which the amplitude were above two times the standard deviation (SD) were considered for the analysis (Additional file 1: Figure S1) [27]. In this way, calcium events were defined from the binary matrix *N* × *F*; where *N* denotes the number of active neurons and *F* the number of frames (1 represents an active and 0 represents an inactive neuron). For each event occurring in a certain cell (trigger neuron), the number of events occurring in a peri-event window (200 and 600 ms) was computed for all the simultaneously imaged cells. Thus, the occurrence probability of synchronized events between CS dorsal horn-projecting, CS intermediate zone projecting and non-identified neurons was obtained.

Additionally, Pearson correlation coefficient from the calcium signals was computed between all pairs of hodologically identified corticospinal neurons. The graphic representation of these coefficients was performed with the open access software Gephi (v 0.9.2).

### Statistics

The number of synchronized calcium events (events occurring in a peri-event window between all pairs of identified neurons) were computed and compared using Kruskal–Wallis ANOVA followed by a Dunn’s test. Additionally, the fraction of synchronized calcium events (events occurring in a peri-event window/total number of events) occurring in CS neurons of the same (DH-DH or IVH-IVH) or in different (DH-IVH or IVH-DH) class were compared with One-way ANOVA followed by Tukey test. Differences were considered significant starting at *P* = 0.05.

## Results

### Proportion of DH- and IVH-projecting CS neurons

**Fig. 1:**
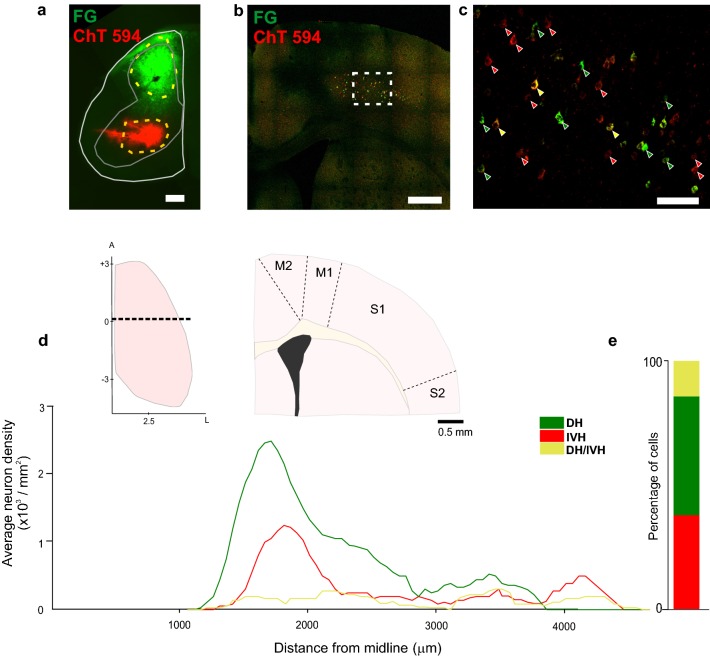
Distribution of CS neurons projecting to DH and IVH in the sensorimotor cortex. **a** Photomicrography of the injection sites of the retrograde tracers (cholera toxin subunit B conjugated with Alexa 594 in red and FluoroGold in green) into the contralateral dorsal horn as well as the intermediate zone and ventral horn of cervical segment C4 in one experiment. Calibration bar 500 μm. **b** Example image from coronal section of the sensorimotor cortex showing the retrogradely-labeled CS neurons projecting to DH (red) and ventral zones (green) in the same experiment. Calibration bar 500 μm. **c** Magnification of the area indicated by the square in **b** showing FG (green arrows) and ChT (red arrows) retrograde labeled neurons. Notice also double-labeled cells in which both retrograde tracers co-localized (yellow arrows). Calibration bar 100 μm. **d** Profile of the distribution of retrograde labeled neurons computed for 3 experiments (3 consecutive slices per experiment) in coronal sections. The upper schemes indicate top and coronal views of the zone from which the neurons are quantified. Dashed line in the top brain view indicated the region of the coronal scheme. Numbers indicates mm from bregma and midline; A, anterior; L, lateral. **e** Proportion of neurons projecting to DH, IVH as well as double labeled cells

**Fig. 2:**
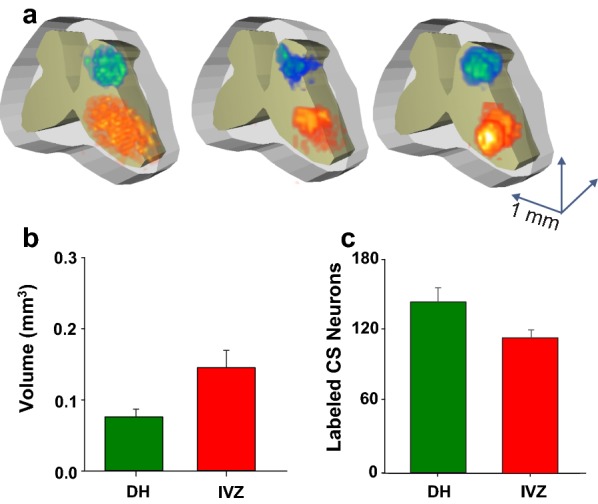
Injection sites of the retrograde tracers into the DH and IVZ. **a** 3D reconstruction of the injection sites in 3 different experiments. Injection sites were performed in C5–C5 spinal cord segments. **b** Volume of tissue labeled with the retrograde tracers injected into the DH and IVZ. **c** Total number of labeled cells counted in 3 consecutive slices in sensorimotor cortex in the same experiments

The relative number of L5 CS neurons projecting to the DH as well as in ventral areas (intermediate gray matter and ventral horn) of the same spinal segment in the sensorimotor cortex was determined in 3 animals. To do so, we simultaneously injected the retrograde neuronal tracers FG and ChT into the cervical (C4–C5) DH and ventral areas (IVH), respectively (Fig. 1a). Only experiments with similar injection sites (location and distribution) were analyzed (Fig. 2). First, we analyzed the distribution of retrogradely labeled neurons in the contralateral sensorimotor cortex from coronal brain slices. The relative number of retrogradely labeled corticospinal FG-positive neurons with respect to ChT-positive neurons was quantified in 3 consecutive slices per experiment (n = 3; 9 slices) in the sensorimotor cortex (Fig. 1). As we previously reported in the rat [23], both groups of neurons (DH- and IVH-projecting) are intermingled around 0.1 mm from bregma in areas corresponding to M1 and S1 (Fig. 1b–d). The proportion of DH and IVH-projecting neurons was 50.0 ± 7.6% (145.3 ± 11.8, n = 3) and 26.8 ± 4.8% (115.6 ± 6.8, n = 3), respectively. Interestingly, the number of double-labeled neurons represents only 13.6 ± 3.7% of the total labeled CS neurons (Fig. 2). No differences were found neither in the soma size (DH-projecting 14.09 ± 0.6 μm; IVZ-projecting 15.5 ± 0.1 μm) nor the distance between different class of CS neurons (DH-DH 109.5 ± 59.5 μm; IVZ-IVZ 94.7 ± 50.5 μm; DH-IVZ 97.8 ± 53.1 μm) (Additional file 2: Figure S2).

### Co-activation analysis of CS neurons

Next, we analyzed the ongoing calcium activity of DH-projecting and IVH-projecting CS neurons, as well as non-identified L5 (NIL5) neurons of the sensorimotor cortex (189 unambiguously identified CS and 51 non-identified neurons) obtained from 8 transgenic animals. The neurons exhibited an oscillatory calcium activity with slow frequency components less than 0.5 Hz. The oscillations of different classes of neurons (DH, IVH-projecting and NIL5 neurons) exhibited a similar amplitude and distribution of frequency components. The area under the curve of the power spectra did not show statistical differences between different groups of neurons (One-way ANOVA test; P = 0.15).

**Fig. 3:**
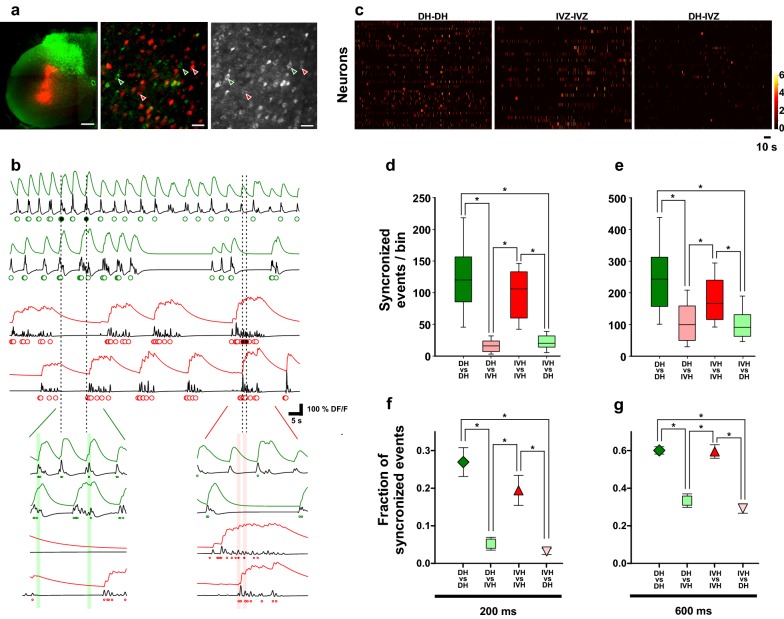
Synchronization between different classes of CS neurons. **a** Photomicrography of the injection sites of the retrograde tracers (cholera toxin subunit B conjugated with Alexa 594 in red and FluoroGold in green) into the contralateral dorsal horn as well as the intermediate zone and ventral horn of cervical segment C4 in one experiment (left) and two-photon microscopy images of a coronal slice showing FG-(green) and ChT-(red) labeled neurons (center), as well as L5 neurons positive to GCaMP6f (right). The field of view corresponds to the area indicated in Fig. 1. **b** (Top) representative traces of simultaneously imaged CS DH- (green) and IVH-projecting (red) neurons indicated by the arrows in **a**. Black traces represent the time derivative of the fluorescence signals. Colored dots indicate the frames (events) in which d(DF/F)/dt remained above the threshold (2 times above the SD of the calcium signal). (Bottom) Zoomed-in depictions of the fluorescence traces indicated in **a**. The shaded areas indicate peri-event intervals triggered by one event occurred in a CS neuron (black dots). Notice that most of the events (dots) occurring during the peri-event interval belong to other CS neurons of the same class (i.e. DH- or IVH-projecting). Thus, for each calcium event occurring in a certain cell, the number of events occurring in a 200-ms or 600-ms peri-event interval (shaded area) of all the simultaneously recorded cells was computed. **c** Color activity maps showing the number of co-activation events (colored scale) between different classes of CS neurons (DH-DH, IVH-IVH and DH-IVH) imaged simultaneously in the same experiment **d**, synchronized events per bin (median, 25th, 75th, 10th and 90th percentiles for all pairs of neurons imaged in 8 experiments) occurring in CS neurons triggered by events in other CS neurons of the same class (between DH-projecting DH-DH; between IVH-projecting IVH-IVH) or in CS neurons of different class (DH-IVH; IVH-DH) computed for 200 ms peri-event interval (*P < 0.05, Kruskal–Wallis ANOVA, post hoc Dunn’s test). **e** the same as C but for the events occurred in 600 ms peri event intervals. (*P < 0.05, Kruskal–Wallis ANOVA, post hoc Dunn’s test). **f**, **g** Fraction of synchronized events (mean ± SE) occurring in CS neurons of the same class (DH-DH and VH-VH) or in CS neurons of different class (DH-IVZ and IVZ-DH) computed for 200 (**e**) and 600 ms (**f**) peri-event intervals (*P < 0.05, One-way ANOVA, post hoc Tukey test)

To characterize the functional synchronization between CS neurons projecting to different zones of the spinal cord, we computed the occurrence probability of synchronized calcium events between different classes of CS neurons. To do so, we discriminated the traces of calcium transients based on a threshold value given by the time derivative [27]. For a certain cell, the number of frames in which DF/F remained above the threshold value was quantified (see “Materials and methods”). Then, we determined the number of events occurring in 2 different peri-event windows (200 and 600 ms), of all the simultaneously imaged cells in the field of view (Fig. 3). In this way, for both peri-event intervals, the difference in the median values of synchronized events per bin in CS DH-projecting or CS IVH-projecting neurons was significantly higher than the number of calcium events between CS neurons projecting to different areas of the spinal cord (Fig. 2c, d) (200 ms time window: H2 = 121.1, P < 0.001 Kruskal–Wallis test, pairwise comparison P < 0.05 Dunn’s test; 600 ms time window: H2 = 1255.9, P = 0.002 Kruskal–Wallis test, pairwise comparison P < 0.05 Dunn’s test). Moreover, the fraction of synchronous events between CS neurons projecting exclusively to one zone was always significantly higher (Fig. 2e, f) than those occurring between CS neurons projecting to different zones (One-way ANOVA test; P ˂ 0.001, pairwise comparison P < 0.05 Tukey test). A correlation between the calcium events rate in the trigger neuron and the number of synchronized events has been observed. However, there is not a significant difference between the ongoing calcium events rate of the different class of CS neurons (Mann–Whitney U test; P > 0.05) (Additional file 2: Figure S2). Moreover, there is not a correlation between the number of synchronized calcium events and Euclidean distance between all pairs of simultaneously imaged identified CS neurons (Additional file 2: Figure S2).

Both population of CS neurons coexist in M1 and medial part of S1 (Fig. 1). Nevertheless, in more rostral cortical areas of M1 and M2 the proportion of CS neurons project mainly to the ventral aspects of the cord. In the caudal and lateral cortical areas corresponding to S1 and S2 the majority of the CS neurons projects to the dorsal horn [23]. In order to analyze in different regions the co-activation properties in other cortical areas, in 8 experiments we analyzed slices from rostral M1 cortical zones (where all the neurons projects to IVZ), as well as caudal S1 cortical areas (where all the neurons projects to DH), comparing co-activation of CS and non-identified neurons (Additional file 3: Figure S3). Interestingly, CS neurons were more synchronized with each other than with non-identified PTNs.

**Fig. 4:**
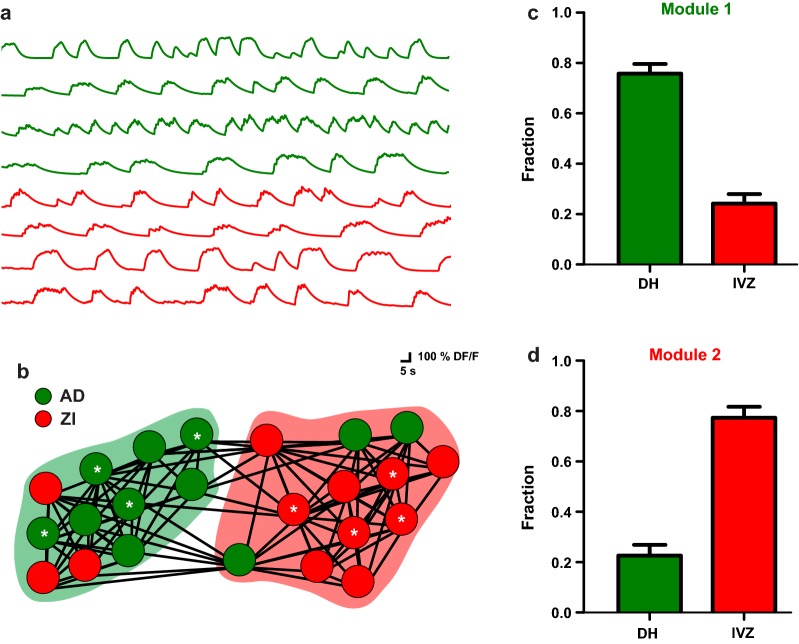
CS neurons projecting to dorsal and ventral horns segregates into distinct functional ensembles. **a** Representative traces of calcium activity for 8 simultaneously imaged CS neurons projecting to DH (green) and IVZ (red). **b** (graphic representation of the correlation of calcium ongoing fluctuations between all the simultaneously imaged neurons projecting to the DH (green) and IVZ (red) obtained in one experiment. Circles represents the imaged neurons and connecting lines represents the positive significant Pearson correlation between elements in the network. The asterisks mark the neurons of the traces in **a**. The shaded areas group the neurons according to the correlation strength into modules based on modularity algorithm proposed by Blondel et al. [28]. Interestingly, in all the experiments (n = 8) two modules has been defined by the algorithm and were named arbitrarily as Module 1 and 2. The graphic representation of the functional networks was performed with the open access software Gephi (v 0.9.2). **c** proportion of neurons projecting to DH and IVZ assigned to the Module 1 computed for all the experiments. **d** The same as **c** but for module 2

In order to compare the connection strength between 2 different populations of subcortically projecting L5 neurons, the temporal correlation degree, between two classes of CS neurons projecting to the dorsal and ventral horn of the same spinal cord segment were analyzed. In this way, we used the significant correlation values, between all pairs of simultaneous imaged neurons, to perform a partition of functional related neuronal ensembles based on the community detection algorithm of modularity optimization [28]. Briefly, the algorithm takes correlation values between all the elements in the functional network and ranks proportionally all interactions to group the elements into communities. Interestingly, in all the experiments (n = 8) two modules has been defined by the algorithm. In this way, we found a significant higher proportion of correlated neurons projecting to the same spinal cord area than the neurons projecting to different zones (Fig. 4), reinforcing that groups of CS neurons segregates into functional ensembles.

## Discussion

Here we show evidence supporting that L5 pyramidal CS neurons in the mouse are segregated into subpopulations projecting to dorsal and ventral aspects in the same segment of the cord. Moreover, CS neurons are organized into distinct, functionally interconnected ensembles. The calcium activity analysis of CS cells indicates that PTNs sharing a determined hodology (i.e., CS DH-projecting and CS IVH-projecting) show synchronized spontaneous activity between each other, suggesting that cortical outputs are hodologically organized into functional segregated ensembles.

We used very small injections of retrograde tracers and only analyzed the experiment in which injection sites were completely separated. In this way, we observed a small proportion of double-labeled cells (Fig. 1). One possible explanation for the double-labeled cells could be that, even though it is not evident, some overlap happened in the retrograde tracer’s injection sites. In fact, in unsuccessful experiments in which the injection of the tracers showed evident overlapping, we observed a large proportion of cells with both tracers, indicating that the existence of double-labeled cells is due to some overlap of the tracers in the injection sites. Our results are in line with previous reports on monkeys [29], cats [30] and rodents [23, 24, 31] in which distinct dorsal and ventral CS projections was observed, suggesting a conserved role of segregated CS projections across species. Nevertheless, it cannot be discarded that the presence of double labeled cells reflects a population of CS neurons broadly branched and bridging different rostrocaudal spinal cord laminae.

Here we used the Thy1-GCaMP6f transgenic mice line GP5.1. Transgenic mice lines expressing fluorescent proteins under the neural-specific promoter of the Thy1 gene have been extensively used for the physiological characterization of projection neocortical and hippocampal pyramidal cells [32–34]. The expression of the Thy1 gene is ubiquitous in the brain and the spinal cord. In the sensorimotor cortex, the expression comprisesL5 corticospinal, corticocollicular and corticothalamic neurons, as well as layer 6 corticothalamic neurons, but not L5 callosal corticocortical neurons and layer 6b corticothalamic neurons [35]. Transgenic lines expressing fluorescent proteins under Thy1 promotor is restricted to cells that express the endogenous gene, with the main differences being the number and subtypes of transgene-positive cells. However, there is a stochastic component to the choice of cells expressing the transgenes [36]. Hence, the expression patterns of Thy1 mouse lines need to be characterized in more detail.

Calcium imaging using genetically encoded calcium indicators is now a standard tool for monitoring activity in neuronal ensembles [37, 38]. Individual spike detection inferred from calcium imaging depends on the type of calcium sensor. In particular, GCaMP6f has probe superiority in spike detection compared with other calcium sensors due to its intrinsic properties, such as fast rise time and decay time [39]. Spike detection efficiency of calcium images depends on the temporal resolution of the images. Sampling at 15 Hz is effective to infer spikes from calcium data [26]. In this study we needed to simultaneously image several L5 pyramidal neurons projecting to different subcortical targets usually contained in a field of view of ~ 400 μm. Thus, we had to sacrifice temporal resolution and acquired the images at 5 Hz. Here, the calcium events produced by a particular neuron were computed from the time derivative and based on a threshold value. Although we did not simultaneously record the electrical activity of the imaged neurons, previous reports have shown that the positive time derivative of the calcium signals is similar to the duration of bursts of action potentials [27].

Here, we perform a co-activation analysis of different classes of CS neurons measuring the occurrence probability of calcium events in peri-events windows (Fig. 3). Because the time series calcium images were collected at 5 Hz, our time window limit for the analysis is 200 ms. In this way, in order to analyze with more detail, the co-activation between CS neurons, we arbitrarily choose another time window for verify the results. In fact, we have the analysis for a time window of 400 ms (data not shown) and similar results were observed.

Our results show that specific subpopulations of CS neurons (i.e. DH-projecting and IVH-projecting) are functionally interconnected within the same group, and they do not connect to the other group (Figs. 3, 4). PTNs are only one subset of pyramidal neurons in L5B; however, intratelencephalic neurons (cortico-cortical and cortico-striatal projecting neurons) are present across all cortical layers [40]. The non-identified L5 neurons in this study are a heterogeneous class containing both intratelencephalic neurons and unlabeled PTNs. Previous studies have shown that PTNs preferentially connect to each other and do not connect to intratelencephalic neurons [41, 42]. Here we show a higher correlated activity between PTNs projecting to one particular zone of the spinal cord compared to other PTNs projecting to different zones, which is consistent with current information about intracortical connectivity. At the microcircuit level, cerebral cortex activity is determined by densely recurrent excitatory connections that generate reverberating activity, linking the neurons into functional neuronal ensembles [37, 38]. Moreover, most cortical neurons belong to largely distributed synaptic circuits receiving and projecting information to numerous other neurons [43]. Actually, the structural and biophysical design of pyramidal neurons seems to specialize in integrating large-scale inputs [3]. Thus, cortical neuron groups or ensembles, rather than individual neurons, are emergent functional units of cortical activity. In this way, the synchronization of different PTN subgroups could be explained by: (i) preferential synaptic connectivity within subsets of L5 projecting neurons, (ii) a higher degree of common inputs and (iii) similar assemblies of calcium channels or means of handling internal calcium stores shared between projecting neuron subgroups. PTNs of the sensorimotor cortex are largely interconnected with other areas of the sensorimotor cortex [44] and with neurons of the different cortical layers in a specific area [41, 42, 45]. The largest synaptic inputs to L5, in both S1 and M1 cortices, are provided by layer 2/3 neurons, but there are also interconnections between different types of L5 neurons, which is more evident in M1 [41, 42], which can explain the higher co-activity in M1 respect to S1 observed here (Additional file 3: Figure S3). In fact, a hierarchical organization between L5 microcircuits has been established, and it is clear that subcortical projection neurons in L5B (i.e., CS neurons) receive unidirectional signaling from higher order corticostriatal neurons [45]. Nevertheless, the sources of synchronization between subsets of L5 neurons in our experimental conditions remain to be determined. Pairwise connectivity studies [41] or optogenetic methods [45] could provide more detailed information about the specific synaptic interactions between different groups of CS neurons.

In addition to the spinal cord, PTNs project to several structures including the posteromedial thalamic nucleus, superior colliculus, pontine nuclei, red nucleus and reticular formation [5–9, 14]. Some evidence supports the segregation of PTNs into different classes depending on the subcortical target [6–9]. However, single neuron reconstructions also show that individual motor cortex PTNs project to multiple subcortical structures [10, 46, 47]. Our results suggest that PTNs are functionally organized into different functional subsystems that may modulate, in a coordinated manner, distinct subcortical circuits associated with different features of sensorimotor control has yet to determine the degree of anatomical segregation in the pyramidal tract system.

L5 PTNs exhibit spontaneous activity in vitro and in vivo [2, 48]. Although their frequency rate varies, they typically fire around 3.5 Hz [9] and show high frequency (≥ 100 Hz) bursts of action potentials [9, 48]. The origin of the spontaneous activity of neuronal ensembles in the central nervous system is not fully understood; however, it is clear that is the spontaneous activity is generated by synaptically-coupled groups of neurons and cooperative properties of the elements in the assemblies [49, 50]. This suggests that the spontaneous synchronous activation of different classes of CS neurons reported in this study is due to common synaptic inputs or reverberant interconnections between a particular class of neurons [38].

The impact of the functional segregation of the CS system remains to be established, although the functional relevance is clear because different classes of interneurons are targets of CS projections and are associated with different aspects of sensorimotor control [24]. Hence, it seems that target-specific intracortical circuits allow PTNs to extract specific features from the same stimulus, which is relayed in parallel to the respective subcortical targets controlling distinct subcortical circuits in a coordinated manner.

## Conclusion

We conclude that distinct groups of CS neurons projecting to dorsal and ventral zones of the transverse cord show synchronous co-activation depending on their hodology, suggesting that CS neurons are organized into different functional ensembles.

## Supplementary information

**Additional file 1: Figure S1.** Identification of individual calcium events. Representative raw fluorescence data from an example neuron (DF/F) and its time derivative (d(DF/F)/dt). Red dashed line indicates the threshold 2.5 times above the SD of the calcium signal. The dots indicate the frames (events) in which d(DF/F)/dt remained above the threshold (peaks above the threshold). Lower traces are zoomed-in depictions of the shaded area indicated in upper traces.

**Additional file 2: Figure S2.** Identification of individual calcium events. **A**, relationship between the number of synchronized calcium events and Euclidean distance between all pairs of simultaneously imaged identified CS neurons. The histogram shows the distribution of the Euclidean distance between different classes of CS neurons (green DH-DH, red IVZ-IVZ, and black DH-IVZ). **B**, relationship between the number of synchronized calcium events and the ongoing calcium events rate in the trigger neuron for all pairs of simultaneously imaged identified CS neurons. The histogram shows the distribution of baseline calcium events rate of the CS neurons projecting to DH (green) and IVZ (red).

**Additional file 3: Figure S3.** Co-activation between CS neurons in different areas of the sensorimotor cortex is higher than non-identified layer 5 cells. **A**, co-activation matrix showing the number of calcium events that simultaneously occurs in all pairs of imaged neurons in motor cortex (M1). The graph bellow shows the fraction of synchronized events (mean ± SE) occurring in CS and non-identified L5 (L5NI) neurons computed in 7 experiments for 200 ms peri-event intervals. **B**, the same as A but for neurons located in somatosensory cortex (S1).

## Data Availability

The datasets used and/or analyzed during the current study are available from the corresponding author on reasonable request.

## Abbreviations

PTNs: pyramidal tract neurons
CS: corticospinal
L5: layer 5
FG: FluoroGold
ChT: cholera toxin subunit b
DH: dorsal horn
IVH: intermediate gray matter and ventral horn
ACSF: artificial cerebrospinal fluid solution
