# Supplementary material for: Mouse corticospinal system comprises different functional neuronal ensembles depending on their hodology
Source: BMC Neurosci. 2019 Sep 23;20:50. doi: 10.1186/s12868-019-0533-5 (PMC6757377; doi:10.1186/s12868-019-0533-5)
Supplement: Supplementary file 1 — Additional file 1: Figure S1. Identification of individual calcium events. Representative raw fluorescence data from an example neuron (DF/F) and its time derivative (d(DF/F)/dt). Red dashed line indicates the threshold 2.5 times above the SD of the calcium signal. The dots indicate the frames (events) in which d(DF/F)/dt remained above the threshold (peaks above the threshold). Lower traces are zoomed-in depictions of the shaded area indicated in upper traces. [file 12868_2019_533_MOESM1_ESM.pdf]

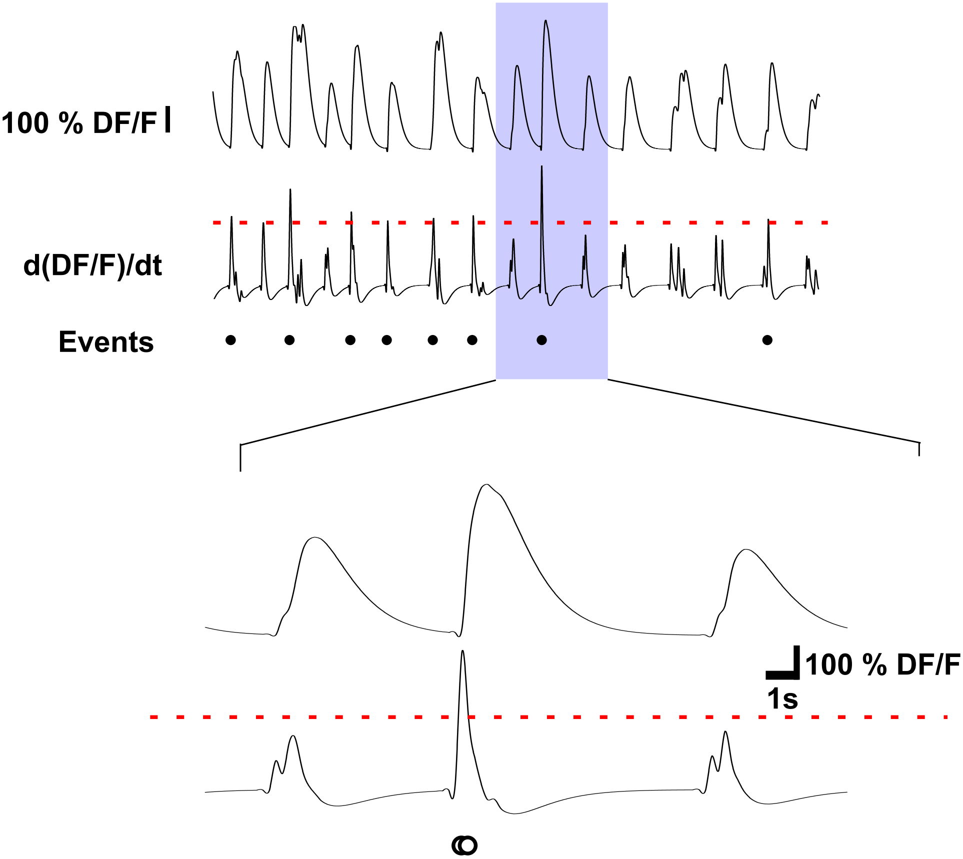

Figure S1. Identification of individual calcium events. Representative raw fluorescence data from an example neuron (DF/F) and its time derivative (d(DF/F)/dt). Red dashed line indicates the threshold 2.5 times above the SD of the calcium signal. The dots indicate the frames (events) in which d(DF/F)/dt remained above the threshold (peaks above the threshold). Lower traces are zoomed-in depictions of the shaded area indicated in upper traces.
