# Supplementary material for: Mouse corticospinal system comprises different functional neuronal ensembles depending on their hodology
Source: BMC Neurosci. 2019 Sep 23;20:50. doi: 10.1186/s12868-019-0533-5 (PMC6757377; doi:10.1186/s12868-019-0533-5)
Supplement: Supplementary file 2 — Additional file 2: Figure S2. Identification of individual calcium events. A, relationship between the number of synchronized calcium events and Euclidean distance between all pairs of simultaneously imaged identified CS neurons. The histogram shows the distribution of the Euclidean distance between different classes of CS neurons (green DH-DH, red IVZ-IVZ, and black DH-IVZ). B, relationship between the number of synchronized calcium events and the ongoing calcium events rate in the trigger neuron for all pairs of simultaneously imaged identified CS neurons. The histogram shows the distribution of baseline calcium events rate of the CS neurons projecting to DH (green) and IVZ (red). [file 12868_2019_533_MOESM2_ESM.pdf]

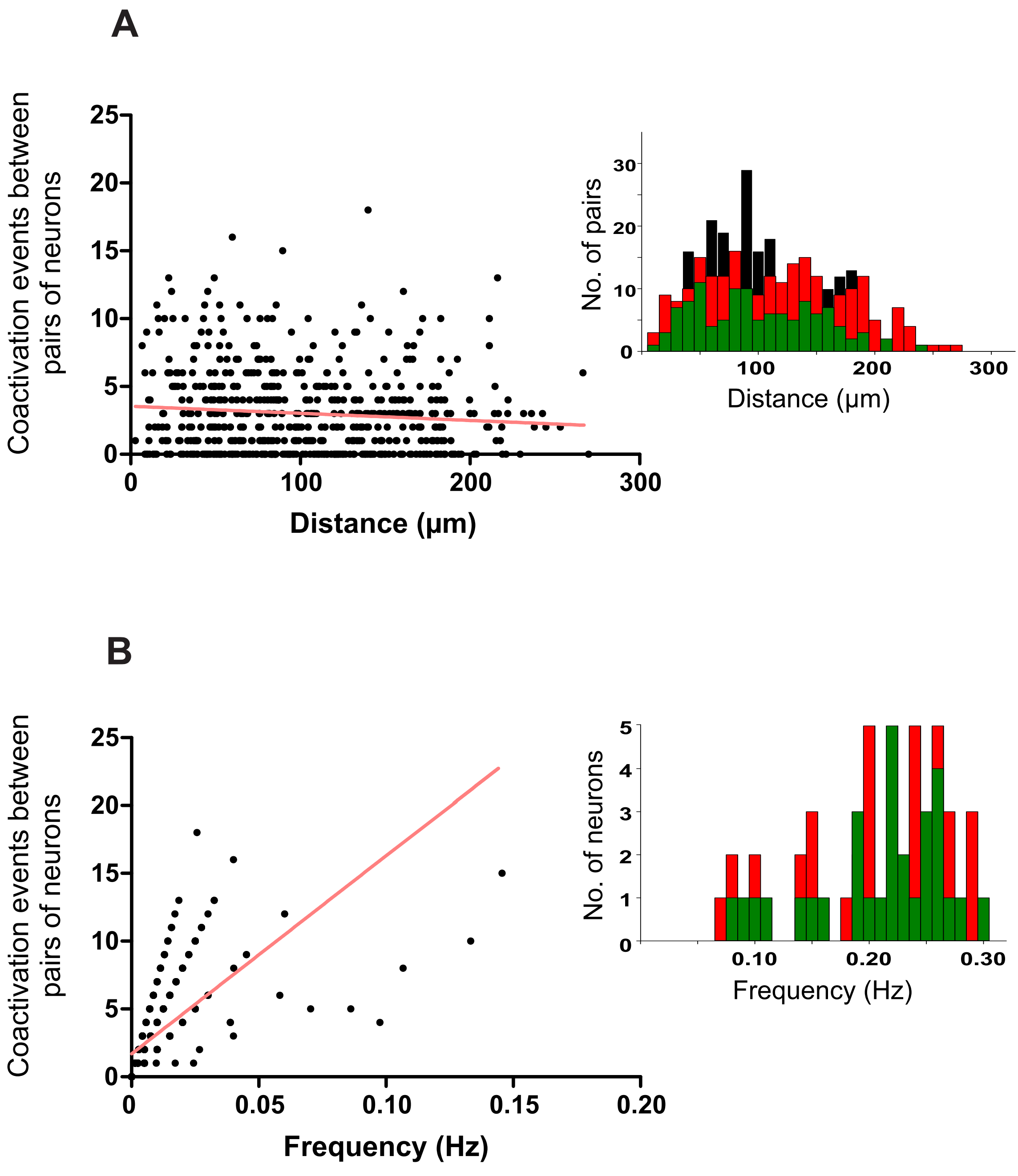

Figure S2. Identification of individual calcium events. A, relationship between the number of synchronized calcium events and Euclidean distance between all pairs of simultaneously imaged identified CS neurons. The histogram shows the distribution of the Euclidean distance between different classes of CS neurons (green DH-DH, red IVZ-IVZ, and black DH-IVZ), **B**, relationship between the number of synchronized calcium events and the ongoing calcium events rate in the trigger neuron for all pairs of simultaneously imaged identified CS neurons. The histogram shows the distribution of baseline calcium events rate of the CS neurons projecting to DH (green) and IVZ (red).
