# Supplementary material for: Mouse corticospinal system comprises different functional neuronal ensembles depending on their hodology
Source: BMC Neurosci. 2019 Sep 23;20:50. doi: 10.1186/s12868-019-0533-5 (PMC6757377; doi:10.1186/s12868-019-0533-5)
Supplement: Supplementary file 3 — Additional file 3: Figure S3. Co-activation between CS neurons in different areas of the sensorimotor cortex is higher than non-identified layer 5 cells. A, co-activation matrix showing the number of calcium events that simultaneously occurs in all pairs of imaged neurons in motor cortex (M1). The graph bellow shows the fraction of synchronized events (mean ± SE) occurring in CS and non-identified L5 (L5NI) neurons computed in 7 experiments for 200 ms peri-event intervals. B, the same as A but for neurons located in somatosensory cortex (S1). [file 12868_2019_533_MOESM3_ESM.pdf]

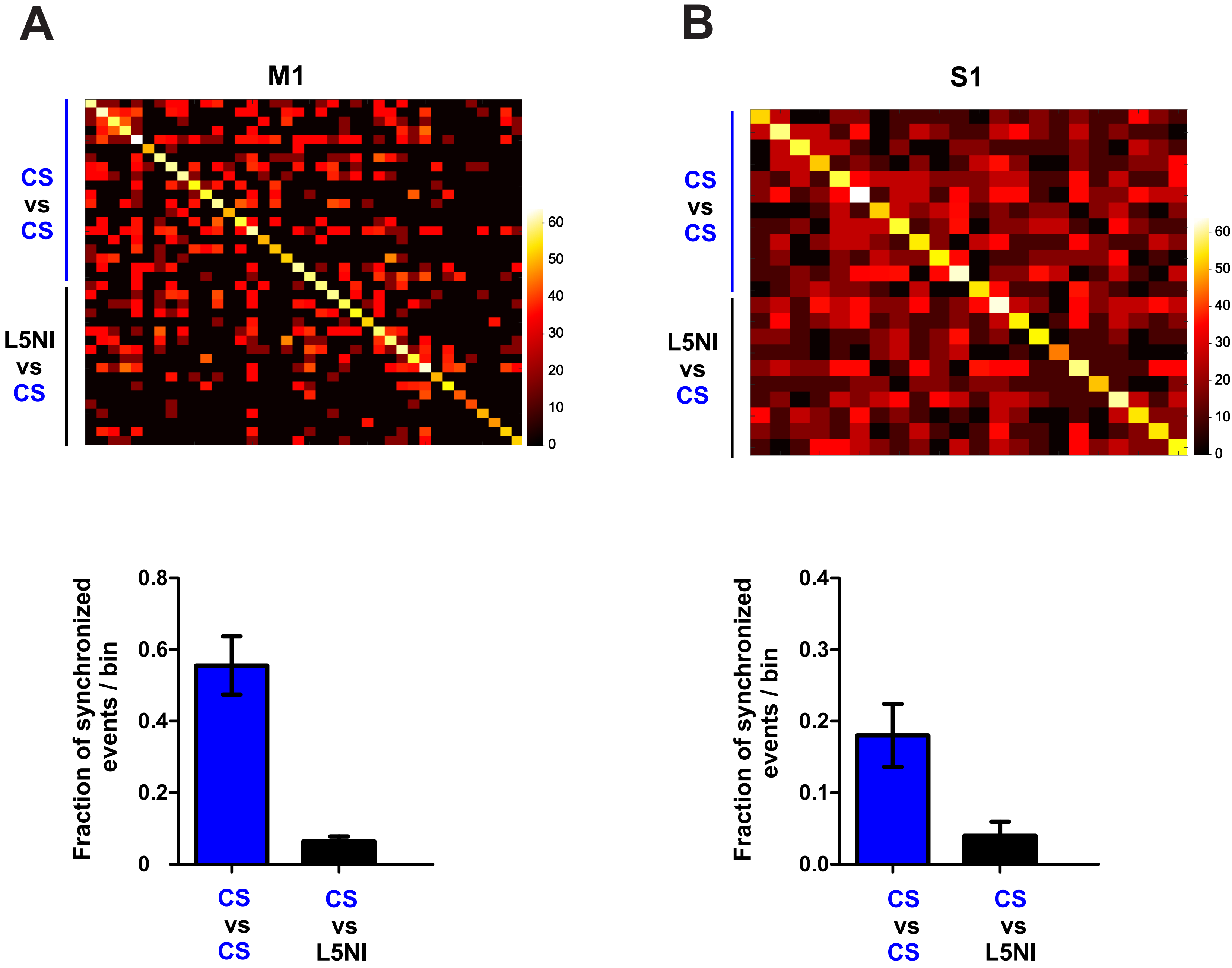

Figure S3. Co-activation between CS neurons in different areas of the sensorimotor cortex is higher than non-identified layer 5 cells. A, co-activation matrix showing the number of calcium events that simultaneously occurs in all pairs of imaged neurons in motor cortex (M1). The graph bellow shows the fraction of synchronized events (mean±SE) occurring in CS and non-identified neurons computed in 7 experiments for 200 ms peri-event intervals. **B**, the same as A but for neurons located in somatosensory cortex (S1).
